# HumanViCe: host ceRNA network in virus infected cells in human

**DOI:** 10.3389/fgene.2014.00249

**Published:** 2014-07-29

**Authors:** Suman Ghosal, Shaoli Das, Rituparno Sen, Jayprokas Chakrabarti

**Affiliations:** ^1^Computational Biology Group, Indian Association for the Cultivation of ScienceKolkata, India; ^2^GyanxetKolkata, India

**Keywords:** virus, microRNA, host-virus interaction, lncRNA, circRNA, ceRNA, immune response, APOBEC

## Abstract

Host-virus interaction via host cellular components has been an important field of research in recent times. RNA interference mediated by short interfering RNAs and microRNAs (miRNA), is a widespread anti-viral defense strategy. Importantly, viruses also encode their own miRNAs. In recent times miRNAs were identified as key players in host-virus interaction. Furthermore, viruses were shown to exploit the host miRNA networks to suite their own need. The complex cross-talk between host and viral miRNAs and their cellular and viral targets forms the environment for viral pathogenesis. Apart from protein-coding mRNAs, non-coding RNAs may also be targeted by host or viral miRNAs in virus infected cells, and viruses can exploit the host miRNA mediated gene regulatory network via the competing endogenous RNA effect. A recent report showed that viral U-rich non-coding RNAs called HSUR, expressed in primate virus herpesvirus saimiri (HVS) infected T cells, were able to bind to three host miRNAs, causing significant alteration in cellular level for one of the miRNAs. We have predicted protein coding and non protein-coding targets for viral and human miRNAs in virus infected cells. We identified viral miRNA targets within host non-coding RNA loci from AGO interacting regions in three different virus infected cells. Gene ontology (GO) and pathway enrichment analysis of the genes comprising the ceRNA networks in the virus infected cells revealed enrichment of key cellular signaling pathways related to cell fate decisions and gene transcription, like Notch and Wnt signaling pathways, as well as pathways related to viral entry, replication and virulence. We identified a vast number of non-coding transcripts playing as potential ceRNAs to the immune response associated genes; e.g., APOBEC family genes, in some virus infected cells. All these information are compiled in HumanViCe (http://gyanxet-beta.com/humanvice), a comprehensive database that provides the potential ceRNA networks in virus infected human cells.

## Introduction

microRNAs (miRNA) are small non coding RNAs (21–24 nucleotides) that play a major role in post-transcriptional gene regulation. Following their discovery in *Caenorhabditis elegans* (Lee et al., 1993; Lau et al., 2001), research on miRNAs progressed rapidly in the past two decades. miRNAs have been identified to be involved in regulation of a variety of cellular processes such as development, differentiation, growth, pluripotency, immune activation, apoptosis, and host-viral interaction (Griffiths-Jones, 2004; Cullen, 2006; German et al., 2008; Zhang and Su, 2008; Bartel, 2009; Xiao and Rajewsky, 2009). As of now, ~24,000 miRNA precursors and ~30,000 mature miRNAs have been annotated from 97 species, and that includes 2042 Homo sapiens mature miRNAs (Griffiths-Jones et al., 2008). miRNAs are known to play a significant role in antiviral defense in most organisms. During viral infection, interaction of viral transcripts or proteins with host cellular components mediates factors like virulence, viral replication, spread of infection, and host immune response (Berkhout and Haasnoot, 2006; Ghosh et al., 2009). It has been observed that cellular miRNAs play important roles on host-viral interaction (Gottwein and Cullen, 2008). Viruses too are found to encode their own miRNAs to exploit host gene silencing machinery (Pfeffer et al., 2004; Dunn et al., 2005; Cui et al., 2006; Nair and Zavolan, 2006; Skalsky et al., 2007; Umbach et al., 2008). Initially the existence of viral miRNAs in Epstein-Barr virus (EBV) was reported by Tuschl group (Pfeffer et al., 2004). Till now, mirBASE lists 295 miRNA genes identified in 27 viruses; amongst them, 56 miRNA genes identified in 11 viruses infecting human cells. These viruses are from the families of Herpesvirus, Polyomaviridae, Adenoviridae, and Retrovirus (Griffiths-Jones et al., 2008). Several virus-encoded miRNAs have been reported to target host transcripts for their own advantage. There are reports of viral miRNAs targeting antiviral signaling molecules, e.g., EBV encoded miR-BHRF1-3 downregulates CXC-chemokine ligand 11 (CXCL11), an interferon (IFN)-inducible T-cell chemoattractant (Xia et al., 2008). The host gene Thrombospondin 1 (THBS1) has been reported to be targeted by multiple KSHV miRNAs, identified through gene expression profiling of cells engineered to stably express 10 KSHV pre-miRNAs (Samols et al., 2007). Another example of cellular mRNA targeted by viral miRNA is PUMA gene targeted by EBV miR-BART5 (Choy et al., 2008). Viral miRNAs may mimic the seed-region sequences of host cellular miRNAs. It has been reported that miR-K12-11 encoded by KSHV, shares the first eight nucleotides with human miRNA-155 (Gottwein et al., 2007; Skalsky et al., 2007) and it is also observed that BACH-1 has been targeted by both human miRNA-155 and KSHV encoded miRNA miR-K-12-11 (Skalsky et al., 2007). Recent studies suggest that cellular miRNA can target other non-coding RNA like long non-coding RNA (lncRNA) and circular RNA (circRNA) (Jeggari et al., 2012; Bhartiya et al., 2013; Ghosal et al., 2013; Hansen et al., 2013; Paraskevopoulou et al., 2013; Li et al., 2014). Moreover, cellular transcripts like mRNAs, pseudogenes, lncRNA, circRNAs, that harbor miRNA response elements (MRE) for one or more common miRNA, can compete with each other for the limited pool of cellular miRNAs and thus affect the competing RNAs level (Sarver and Subramanian, 2012; Li et al., 2014). Competing Endogenous RNAs or ceRNAs have been found to have important roles in a variety of cellular processes like cell cycle control and tumor suppression, e.g., PTEN-P1 blocking miR-19b and miR-20a from binding to PTEN tumor suppressor (Karreth et al., 2011; Sumazin et al., 2011; Tay et al., 2011). ceRNAs can modulate self-regulation in hepatocellular carcinoma, e.g., HULC lncRNA acts as ceRNA of the protein coding gene PRKACB that induces activation of CREB which in turn is involved in upregulation of HULC (Wang et al., 2010). It is also observed that ceRNAs have important role in developmental stages e.g., linc-MD1 blocking miR-133 from binding to transcription factors involved in myogenic differentiation (Cesana et al., 2011) and H19 blocking the miRNA let-7 to affect muscle differentiation *in vitro* (Kallen et al., 2013). Importantly, there is evidence for the viral strategy of exploiting host gene regulatory circuit by ceRNA effect. Cazalla and his group have reported that viral U-rich non-coding RNAs called HSUR expressed in primate virus herpesvirus saimiri (HVS) infected T cells are able to bind to three host miRNAs. They also noted that this activity resulted in striking alteration of the cellular levels of one of these miRNAs, miRNA-27. This phenomenon leads to the regulation of expression of the host-cell genes targeted by this miRNA. Hence, the potential of viral miRNAs to exploit the host gene regulatory network via the ceRNA effect is suggested. As viral miRNAs have already been reported to interact with host cellular factors, they could have potential interaction with cellular non-coding RNAs. We identified potential lncRNA and circRNA targets for both host and viral miRNAs from AGO PAR-CLIP datasets in some virus infected cells including EBV infected lymphoblastoid cell lines (LCLs), Human cytomegalovirus (HCMV) infected primary human fibroblast cells and two latently Kaposi's sarcoma associated herpesvirus (KSHV) infected primary effusion lymphoma (PEL) cell lines, BCBL-1, and BC-3. We developed a repository of the putative viral and host (human) miRNA interaction with cellular protein-coding RNA, lncRNA, and circRNA and modeled the potential ceRNA functions in virus infected cells in human. Gene ontology (GO) and pathway enrichment analysis of the genes comprising the ceRNA networks in the virus infected cells revealed enrichment of key cellular signaling pathways related to cell fate decisions and gene transcription as well as pathways related to viral entry, replication, and virulence. These cellular pathways are known to be frequently manipulated by virus to facilitate their own spreading. Our database HumanViCe provides the users with the potential ceRNA networks in virus infected human cells that are instrumental in fine tuning of gene expressions to aid the host defense against viruses. We believe this database to be an important resource for exploration of the role of host ceRNAs in viral infection.

## Methods

### Sequence data collection

We collected human and viral mature miRNA sequence data from miRBase database (Griffiths-Jones et al., 2008). We collected miRNA sequences from 11 virus species known to infect human. These 11 virus species included Epstein barr virus (EBV), Herpes Simplex Virus 1 (HSV1), Herpes Simplex Virus 2 (HSV2), BK polyomavirus (BKV), HCMV, Human immunodeficiency virus 1 (HIV1), JC polyomavirus miRNAs (JCV), Kaposi sarcoma-associated herpesvirus (KSHV), Merkel cell polyomavirus (MCV), Simian virus 40 (SV40) and Human herpesvirus 6B. Human lncRNAs data were collected from the current version of GENCODE human, GENCODE 19. GENCODE 19 annotated 13220 lncRNA genes (Derrien et al., 2012). The human circRNA dataset consisting of 1953 predicted human circRNAs was collected from the study of Memczak et al (Memczak et al., 2013).

### Prediction of host and viral miRNA targets on host

The dataset for viral miRNA targets on human protein coding transcripts was collected from vHot database where targets were predicted by TargetScan, miRanda, RNAhybrid, microT, and PITA (Kim et al., 2012). We collected putative human miRNA targets on human protein coding transcripts from Targetscan (Lewis et al., 2005).

For prediction of host and viral miRNA targets on human lncRNAs and circRNAs, we developed a custom algorithm for seed matched target finding coupled with favorable duplex stability. For Seed complementarity search, a modified Smith-Waterman algorithm was used and for prediction of different types of miRNA target sites (6-mer, 7-mer, 7-merA1, and 8-mer). We considered transcripts with seed complementarity as well as one base mismatch tolerance (in position 2–8 or 2–7) in the seed region with 3′ compensatory complementarities. To reduce runtime involved in computation of target sites on huge set of transcripts (~25000 lncRNA+circRNA), we first searched for sites containing perfect 6-mer complementarity with miRNA seed from either miRNA 5′ end (2–7th base) or 3′ end (13–18th base). In case a match was found, then a 25 base window around the seed-matched site (starting from two base preceding seed-matched site in case of miRNA 5′ end seed match, starting from 14 base preceding seed-matched site in case of miRNA 3′ end seed match) was used for further alignment by the modified Smith-Waterman algorithm.

For calculating duplex energy of the predicted miRNA-target duplex we used the Vienna RNA package (Hofacker et al., 1994). We calculated target accessibility for each miRNA-target by the following:

(1) $$E_{d}=E_{mil}-E_{ln}<-20\text{kcal/mol},$$

where,

*E_mil_* = Duplex energy of the putative miRNA-target pair (calculated using the cofold routine in Vienna RNA package)

*E_ln_* = Free energy of the target lncRNA (calculated using the fold routine in Vienna RNA package).

### Validation of miRNA-lncRNA interaction predicted by our custom algorithm

For partial validation and providing a measure of accuracy in our prediction of miRNA targets on host lncRNAs, we verified our miRNA target finding program on the dataset of Nam et al NCBI GEO accession no. GSE52530 (Nam et al., 2014). This dataset consisted of RNA-Seq expression profiling of cellular transcripts in four different cell lines (HeLa, HEK293, Huh7, and IMR90) after transfection of miR-124 and miR-155 respectively, compared to mock transfection. The transcripts included non-coding transcripts also (with RefSeq annotation prefix NR_). From the dataset of HEK293 cells, out of 4789 non-coding (NR_) transcripts, 1617 and 1552 transcripts showed decreased expression (log 2 fold change<-1) upon transfection of miR-124 and miR-155 respectively compared to the mock transfected control. The log 2 fold changes were calculated using the R package foldchange and foldchange2logratio. We regarded these 1617 and 1552 non-coding transcripts as positive control for miR-124 and miR-155 respectively and the rest (3172 and 3537 transcripts) were regarded as negative control. From these two datasets, we predicted targets of miR-124 and miR-155 respectively to measure the accuracy of our algorithm in terms of false discovery rate (FDR). FDR measures the specificity of an algorithm in terms of its false positive detections.

FDR = FP/FP+TN, where FP = number of false positives and TN = number of true positives.

From this dataset, our algorithm detected only 95 false positives (FP) out of a total of 2363 negative sets (FP+TN), which gave an FDR of 0.04. The low FDR was due to the stringency of the algorithm that required perfect seed matches (either from miRNA 5′ end or 3′ end) and a filtering for favorable miRNA-target duplex. Though the stringent conditions resulted in much false negative detection in the current dataset, we kept these conditions to ensure a low false positive rate that was particularly required for the present work.

### Collection of miRNA expression data in virus infected cells

We used host (human) miRNA expression profiling data from EBV, HCMV, HIV1, and KSHV infected cells, collected from NCBI GEO database. miRNA expression profile from small RNA deep sequencing of EBV B95.8-infected LCLs was collected from GEO series accession no. GSE41437, high-throughput profiling of smRNAs, Ago1, and Ago2-associated miRNAs from HCMV-infected fibroblast cells was collected from GSE33584, miRNA expression profiles in the peripheral blood mononuclear cells (PBMCs) after HIV1 infection was collected from GSE44332, Ago HITS-CLIP in KSHV-infected PEL cell lines BCBL-1 and BC-3 was collected from GSE41357.

### Calculating the probability of ceRNA pair to cross-regulate each other

We implemented a measure to assess the likelihood of a ceRNA pair to regulate each other via shared miRNAs. This approach was similar to what has been used in the study of Sumazin et al. (2011) and in StarBase v2.0 (Li et al., 2014). We calculated the *p*-value for each potential ceRNA pair by hypergeometric test considering the number of shared miRNAs between a ceRNA pair against the number of miRNAs targeting individual components of the ceRNA pair. The *p*-value was measured as:

(2) $$p=\sum_{i=m_{c}}^{min\left(m_{p},m_{n}\right)}\frac{\left(\begin{array}{c} m_{n}\\ i \end{array}\right)\left(\begin{array}{c} M_{T}-m_{n}\\ m_{p}-i \end{array}\right)}{\left(\begin{array}{c} M_{T}\\ m_{p} \end{array}\right)},$$

where,

*M_T_* = Total number of miRNAs in the human genome

*m_p_* = Number of miRNAs interacting with the first ceRNA

*m_n_* = Total number of miRNAs interacting with the second ceRNA

*m_c_* = Number of miRNAs shared between the ceRNA pair.

### Implementation

**Figure 1:**
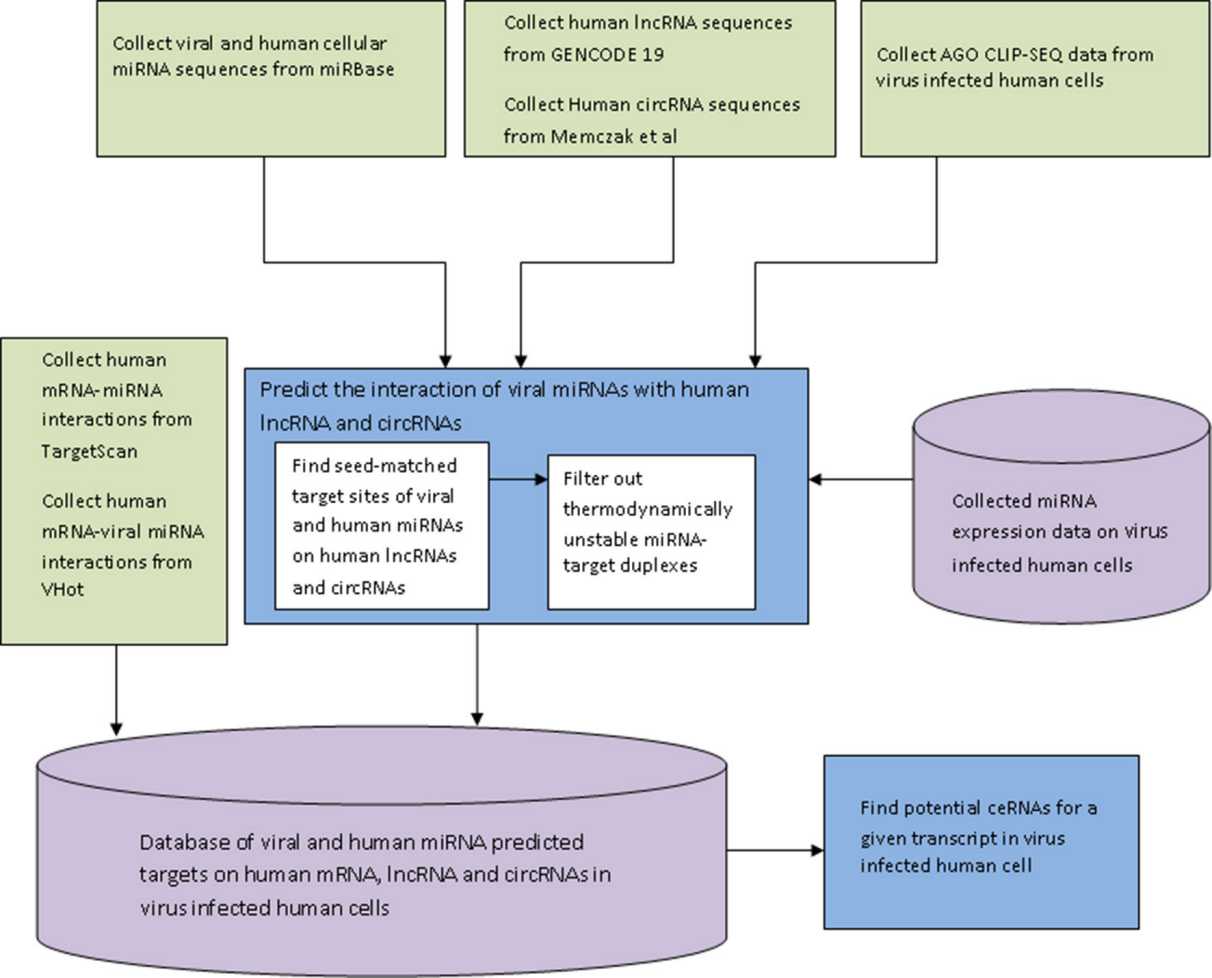
**The flow-diagram of the steps involved in development of the database HumanViCe**. The predicted interaction of the host miRNA-host target and viral miRNA-host target was collected from Targetscan and VHot databases respectively. The interaction of host lncRNA and circRNA with viral miRNA was predicted by our custom algorithm. We also stored the miRNA expression profiling data from virus infected cells collected from NCBI GEO (GSE41437, GSE33584, GSE44332, and GSE41357). All the miRNA target interaction data (host miRNA-host target and viral miRNA-host target), along with miRNA expression profiles are stored in a mySQL database which can now be queried for potential ceRNAs of a transcript in a given virus infected cell.

The miRNA target finding algorithm was implemented in JAVA and the web interface of the database HumanViCe was developed using PHP-mySql. The collected dataset of viral miRNA-host mRNA interactions and host miRNA-host miRNA interactions along with our generated dataset of viral miRNA-host lncRNA and circRNA and host miRNA-host lncRNA interactions were stored in a mySQL database. The dataset of host miRNA expression profiles in virus infected cells were also stored in the database (for EBV, HIV1, HCMV and KSHV). For these four viruses, only the targets of host miRNAs expressed in the virus infected cells were displayed. Browsing of the database HumanViCe for viral miRNA targets or ceRNAs of a target transcript by users is enabled by mySql queries from PHP. Figure 1 shows the flowchart for development of the database HumanViCe.

## Results

### Database contents

Presently HumanViCe contains targets for a total of 144 viral miRNAs encoded by 10 viruses known to infect human cells (see Table 1). Other than the viral miRNA targets, HumanViCe also lists host cellular miRNA targets to present a more comprehensive picture of the miRNA mediated regulations in virus infected cells. Like host cellular miRNAs, viral miRNAs were also predicted to target host non-coding transcripts like lncRNA and circRNAs. From our prediction, 144 viral miRNAs were found to potentially target 6257 human lncRNA transcripts (from the set of 23898 annotated lncRNA transcripts in GENCODE 19 version) resulting in 10262 putative host lncRNA-viral miRNA interactions. Viral miRNAs were also found to potentially target 1277 human circRNA transcripts among the set of 1954 circRNA candidates from the study of Memczak et al. (2013). As previously reported, these non-coding transcripts bearing MREs can act as miRNA sponge to regulate the availability of the targeting miRNAs. Especially circRNAs are reported to contain extensive miRNA binding sites for effective miRNA sponge activity. From our study we identified some circRNAs containing large number of binding sites for some viral miRNAs, e.g., human circRNA transcript of ANKRD11 (circRNA id: hsa_circ_002048) was found to bear putative 36 sites for EBV encoded miRNA ebv-miR-BART20-5p.

**Table 1:** **miRNAs encoded by viruses known to infect human**.

**Virus**	**Encoded miRNAs**
Epstein Barr Virus	ebv-miR-BART4-3p CACAUCACGUAGGCACCAGGUGU
	ebv-miR-BART20-3p CAUGAAGGCACAGCCUGUUACC
	ebv-miR-BHRF1-2-5p AAAUUCUGUUGCAGCAGAUAGC
	ebv-miR-BART22 UUACAAAGUCAUGGUCUAGUAGU
	ebv-miR-BART14-3p UAAAUGCUGCAGUAGUAGGGAU
	ebv-miR-BART10-3p UACAUAACCAUGGAGUUGGCUGU
	ebv-miR-BART7-3p CAUCAUAGUCCAGUGUCCAGGG
	ebv-miR-BART16 UUAGAUAGAGUGGGUGUGUGCUCU
	ebv-miR-BART15 GUCAGUGGUUUUGUUUCCUUGA
	ebv-miR-BART9-3p UAACACUUCAUGGGUCCCGUAGU
	ebv-miR-BART21-5p UCACUAGUGAAGGCAACUAAC
	ebv-miR-BART3-3p CGCACCACUAGUCACCAGGUGU
	ebv-miR-BART7-5p CCUGGACCUUGACUAUGAAACA
	ebv-miR-BART1-3p UAGCACCGCUAUCCACUAUGUC
	ebv-miR-BART11-5p UCAGACAGUUUGGUGCGCUAGUUG
	ebv-miR-BART6-3p CGGGGAUCGGACUAGCCUUAGA
	ebv-miR-BART13-5p AACCGGCUCGUGGCUCGUACAG
	ebv-miR-BART1-5p UCUUAGUGGAAGUGACGUGCUGUG
	ebv-miR-BART2-5p UAUUUUCUGCAUUCGCCCUUGC
	ebv-miR-BART2-3p AAGGAGCGAUUUGGAGAAAAUAAA
	ebv-miR-BHRF1-3 UAACGGGAAGUGUGUAAGCACA
	ebv-miR-BART14-5p UACCCUACGCUGCCGAUUUACA
	ebv-miR-BART18-5p UCAAGUUCGCACUUCCUAUACA
	ebv-miR-BART4-5p GACCUGAUGCUGCUGGUGUGCU
	ebv-miR-BART8-3p GUCACAAUCUAUGGGGUCGUAGA
	ebv-miR-BHRF1-2-3p UAUCUUUUGCGGCAGAAAUUGA
	ebv-miR-BART20-5p UAGCAGGCAUGUCUUCAUUCC
	ebv-miR-BART13-3p UGUAACUUGCCAGGGACGGCUGA
	ebv-miR-BART19-3p UUUUGUUUGCUUGGGAAUGCU
	ebv-miR-BART8-5p UACGGUUUCCUAGAUUGUACAG
	ebv-miR-BART5-5p CAAGGUGAAUAUAGCUGCCCAUCG
	ebv-miR-BART17-3p UGUAUGCCUGGUGUCCCCUUAGU
	ebv-miR-BART17-5p UAAGAGGACGCAGGCAUACAAG
	ebv-miR-BHRF1-1 UAACCUGAUCAGCCCCGGAGUU
	ebv-miR-BART19-5p ACAUUCCCCGCAAACAUGACAUG
	ebv-miR-BART18-3p UAUCGGAAGUUUGGGCUUCGUC
	ebv-miR-BART6-5p UAAGGUUGGUCCAAUCCAUAGG
	ebv-miR-BART12 UCCUGUGGUGUUUGGUGUGGUU
	ebv-miR-BART21-3p CUAGUUGUGCCCACUGGUGUUU
	ebv-miR-BART3-5p ACCUAGUGUUAGUGUUGUGCU
	ebv-miR-BART5-3p GUGGGCCGCUGUUCACCU
	ebv-miR-BART9-5p UACUGGACCCUGAAUUGGAAAC
	ebv-miR-BART10-5p GCCACCUCUUUGGUUCUGUACA
	ebv-miR-BART11-3p ACGCACACCAGGCUGACUGCC
Herpes Simplex Virus 1	hsv1-miR-H8-5p UAUAUAGGGUCAGGGGGUUC
	hsv1-miR-H13 UUAGGGCGAAGUGCGAGCACUGG
	hsv1-miR-H1-5p GAUGGAAGGACGGGAAGUGGA
	hsv1-miR-H6-5p GGUGGAAGGCAGGGGGGUGUA
	hsv1-miR-H11 UUAGGACAAAGUGCGAACGC
	hsv1-miR-H14-3p UCUGUGCCGGGCGCGUGCGAC
	hsv1-miR-H7-5p AAAGGGGUCUGCAACCAAAGG
	hsv1-miR-H26 UGGCUCGGUGAGCGACGGUC
	hsv1-miR-H7-3p UUUGGAUCCCGACCCCUCUUC
	hsv1-miR-H8-3p GCCCCCGGUCCCUGUAUAUA
	hsv1-miR-H6-3p CACUUCCCGUCCUUCCAUCCC
	hsv1-miR-H1-3p UACACCCCCCUGCCUUCCACCCU
	hsv1-miR-H2-3p CCUGAGCCAGGGACGAGUGCGACU
	hsv1-miR-H3-3p CUGGGACUGUGCGGUUGGGAC
	hsv1-miR-H4-5p GGUAGAGUUUGACAGGCAAGCA
	hsv1-miR-H17 UGGCGCUGGGGCGCGAGGCGG
	hsv1-miR-H14-5p AGUCGCACUCGUCCCUGGCUCAGG
	hsv1-miR-H4-3p CUUGCCUGUCUAACUCGCUAGU
	hsv1-miR-H16 CCAGGAGGCUGGGAUCGAAGGC
	hsv1-miR-H5-5p GGGGGGGUUCGGGCAUCUCUAC
	hsv1-miR-H2-5p UCGCACGCGCCCGGCACAGACU
	hsv1-miR-H12 UUGGGACGAAGUGCGAACGCUU
	hsv1-miR-H18 CCCGCCCGCCGGACGCCGGGACC
	hsv1-miR-H15 GGCCCCGGGCCGGGCCGCCACG
	hsv1-miR-H5-3p GUCAGAGAUCCAAACCCUCCGG
	hsv1-miR-H3-5p CUCCUGACCGCGGGUUCCGAGU
Herpes Simplex Virus 2	hsv2-miR-H11-3p UUAGGACAAAGUGCGAACGCUU
	hsv2-miR-H7-5p AAAGGGGUCCGUAACCAAAGG
	hsv2-miR-H23-3p ACGAGCUUCGCGGUACUACUC
	hsv2-miR-H19 UUCGCUAGGCAAGCACGGACUG
	hsv2-miR-H4-5p GAGUUCACUCGGCACGCAUGC
	hsv2-miR-H3 UUUGGGAGUCUGCGGUUGGGAG
	hsv2-miR-H20 UUUGGUUACGGACCCCUUUCU
	hsv2-miR-H21 AUAACGUCAUGCUGUCUACGG
	hsv2-miR-H9-3p UUCCCACCUCGGUCUCCUCCUC
	hsv2-miR-H6-3p CCCAUCUUCUGCCCUUCCAUCCU
	hsv2-miR-H23-5p AGGCCGUGGAGCUUGCCAGC
	hsv2-miR-H7-3p UUUGGAUUCCGACCCCUCGUC
	hsv2-miR-H11-5p AAGCGUUCGCACUUUGUCCUA
	hsv2-miR-H5 GGGGGGGCUCGGGCCACCUGACC
	hsv2-miR-H4-3p CCGUGCUUGCCUAGCGAACUC
	hsv2-miR-H10 GGGUGCGGGGGUGGGCGG
	hsv2-miR-H22 AGGGGUCUGGACGUGGGUGGGC
	hsv2-miR-H25 CUGCGCGGCGGAGACCGGGAC
	hsv2-miR-H13 UUAGGGCAAAGUGCGAGCACUG
	hsv2-miR-H2 UCUGAGCCUGGGUCAUGCGCGA
	hsv2-miR-H9-5p CUCGGAGGUGGAGUCGCGGU
	hsv2-miR-H6-5p AAUGGAAGGCGAGGGGAUGC
	hsv2-miR-H12 UUAGGACGAAGUGCGAACGCUU
	hsv2-miR-H24 CUCCGGCGCCUUCCCCCCGCCCU
BK	bkv-miR-B1-3p UGCUUGAUCCAUGUCCAGAGUC
Polyomavirus	bkv-miR-B1-5p AUCUGAGACUUGGGAAGAGCAU
Human cytomegalovirus	hcmv-miR-US25-1-3p UCCGAACGCUAGGUCGGUUCUC
	hcmv-miR-US25-2-3p AUCCACUUGGAGAGCUCCCGCGG
	hcmv-miR-UL36-3p UUUCCAGGUGUUUUCAACGUGC
	hcmv-miR-US4 CGACAUGGACGUGCAGGGGGAU
	hcmv-miR-UL70-3p GGGGAUGGGCUGGCGCGCGG
	hcmv-miR-US25-2-5p AGCGGUCUGUUCAGGUGGAUGA
	hcmv-miR-UL70-5p UGCGUCUCGGCCUCGUCCAGA
	hcmv-miR-UL22A-5p UAACUAGCCUUCCCGUGAGA
	hcmv-miR-UL36-5p UCGUUGAAGACACCUGGAAAGA
	hcmv-miR-US25-1-5p AACCGCUCAGUGGCUCGGACC
	hcmv-miR-UL112 AAGUGACGGUGAGAUCCAGGCU
	hcmv-miR-US33-5p GAUUGUGCCCGGACCGUGGGCG
	hcmv-miR-UL148D UCGUCCUCCCCUUCUUCACCG
	hcmv-miR-US5-2 UUAUGAUAGGUGUGACGAUGUC
	hcmv-miR-US5-1 UGACAAGCCUGACGAGAGCGU
	hcmv-miR-UL22A-3p UCACCAGAAUGCUAGUUUGUAG
	hcmv-miR-US33-3p UCACGGUCCGAGCACAUCCA
Human Immunodeficiency virus	hiv1-miR-H1 CCAGGGAGGCGUGCCUGGGC
	hiv1-miR-N367 ACUGACCUUUGGAUGGUGCUUCAA
	hiv1-miR-TAR-3p UCUCUGGCUAACUAGGGAACCCA
	hiv1-miR-TAR-5p UCUCUCUGGUUAGACCAGAUCUGA
JC polyomavirus	jcv-miR-J1-3p UGCUUGAUCCAUGUCCAGAGUC
	jcv-miR-J1-5p UUCUGAGACCUGGGAAAAGCAU
Kaposi's sarcoma associated herpesvirus	kshv-miR-K12-5-3p UAGGAUGCCUGGAACUUGCCGGU
	kshv-miR-K12-4-3p UAGAAUACUGAGGCCUAGCUGA
	kshv-miR-K12-8-5p ACUCCCUCACUAACGCCCCGCU
	kshv-miR-K12-10a-3p UAGUGUUGUCCCCCCGAGUGGC
	kshv-miR-K12-5-5p AGGUAGUCCCUGGUGCCCUAAGG
	kshv-miR-K12-8-3p CUAGGCGCGACUGAGAGAGCA
	kshv-miR-K12-6-3p UGAUGGUUUUCGGGCUGUUGAG
	kshv-miR-K12-6-5p CCAGCAGCACCUAAUCCAUCGG
	kshv-miR-K12-3-5p UCACAUUCUGAGGACGGCAGCGA
	kshv-miR-K12-2-5p AACUGUAGUCCGGGUCGAUCUG
	kshv-miR-K12-12-3p UGGGGGAGGGUGCCCUGGUUGA
	kshv-miR-K12-11-5p GGUCACAGCUUAAACAUUUCUAGG
	kshv-miR-K12-1-3p GCAGCACCUGUUUCCUGCAACC
	kshv-miR-K12-9-3p CUGGGUAUACGCAGCUGCGUAA
	kshv-miR-K12-12-5p AACCAGGCCACCAUUCCUCUCCG
	kshv-miR-K12-10b UGGUGUUGUCCCCCCGAGUGGC
	kshv-miR-K12-3-3p UCGCGGUCACAGAAUGUGACA
	kshv-miR-K12-11-3p UUAAUGCUUAGCCUGUGUCCGA
	kshv-miR-K12-1-5p AUUACAGGAAACUGGGUGUAAGC
	kshv-miR-K12-9-5p ACCCAGCUGCGUAAACCCCGCU
	kshv-miR-K12-2-3p GAUCUUCCAGGGCUAGAGCUG
	kshv-miR-K12-7-5p AGCGCCACCGGACGGGGAUU
Merkel cell polyomavirus	mcv-miR-M1-5p UGGAAGAAUUUCUAGGUACACU
	mcv-miR-M1-3p UGUGCUGGAUUCUCUUCCUGAA
Simian virus 40	sv40-miR-S1-3p GCCUGUUUCAUGCCCUGAGU
	sv40-miR-S1-5p UGAGGGGCCUGAAAUGAGCCUU

### Analysis of the predicted host ceRNA networks for host-virus interaction

**Figure 2:**
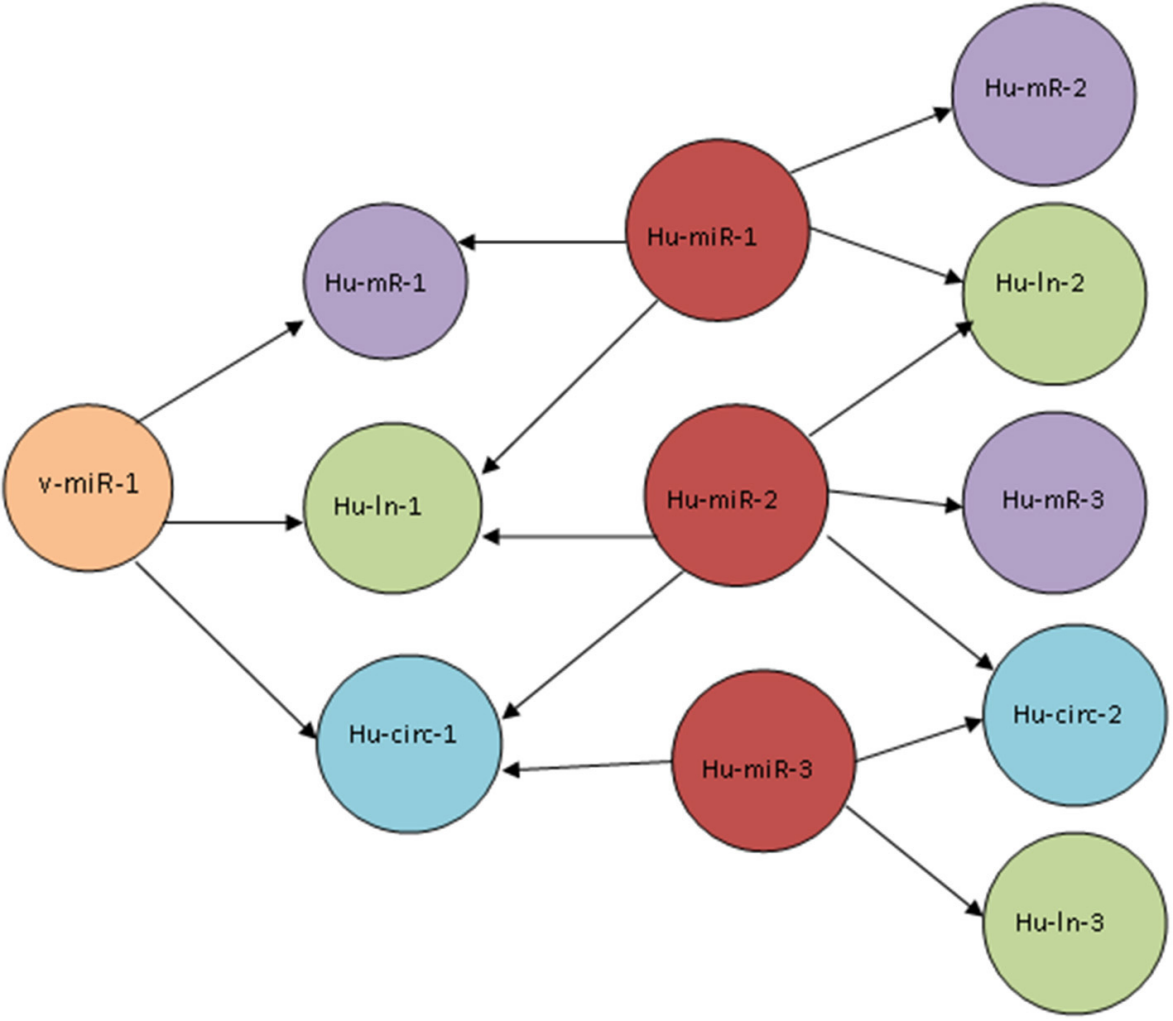
**Schematic diagram of the connections in a host-virus ceRNA network**. A viral miRNA (v-miR-1) targets three cellular transcripts (Hu-mR-1, Hu-ln-1, and Hu-circ-1). These transcripts are also targeted by host miRNAs Hu-miR-1, Hu-miR-2, and Hu-miR-3. The cellular transcripts Hu-mR-2, Hu-mR-3, Hu-ln-2, Hu-ln-3 and Hu-circ-2 do not have a direct connection with the viral miRNA but shares Hu-miR-1,2, and 3 with Hu-mR-1, Hu-ln-1, and Hu-circ-1 and thus they are connected by a ceRNA network.

We built the predicted ceRNA network for host-virus interaction for 10 viruses which are known to infect human and which encode viral miRNAs (see Table 1). These ceRNA networks consist of host mRNAs, lncRNAs and circRNAs sharing common viral miRNAs or host miRNAs. Figure 2 shows a schematic diagram that depicts the typical connections in such a ceRNA network. To get an insight into the biological significance of these ceRNA networks for host-virus interactions, we performed GO enrichment analysis by GORILLA (Eden et al., 2009) and pathway enrichment analysis by KOBAS 2.0 (Xie et al., 2011) using pathway databases KEGG pathway and Reactome. Interestingly, when we looked into the top 20 most enriched pathways (*p*-value< 0.05) for ceRNA networks corresponding to each virus, we found many common pathways to be enriched in all these networks. The most common enriched pathways include Axon Guidance (for all 10 viruses), signaling by NGF (all viruses except JCV), Hippo signaling pathway (all viruses except SV40), MAPK signaling pathway (all viruses except SV40), signaling by NOTCH (all viruses except JCV and SV40), Proteoglycans in cancer (all viruses except HSV2 and KSHV), Wnt signaling pathway (all viruses except HCMV, JCV, and SV40). The key cellular signaling pathways related to cell fate decisions and gene transcription, like Notch signaling and Wnt signaling pathways are frequently utilized or manipulated by viruses to suite their own need (Hayward, 2004; Shackelford and Pagano, 2004). Our observation indicates that viruses may exploit these pathways also via the ceRNA network around them. Moreover, the GO process and pathway enrichment analysis of the ceRNA networks for all the viruses revealed enrichment of pathways related to viral entry, replication and virulence. The genes in the ceRNA networks of EBV, HCMV, HIV1, HSV1, HSV2, and KSHV were found to be enriched for endocytosis. The genes in the ceRNA networks of EBV, HIV1, HSV1, HSV2, and KSHV were found to be enriched for Membrane trafficking (Tokarev and Guatelli, 2011). This observation may provide further insight into viral strategy as viruses like HIV are already known to manipulate the intracellular membrane trafficking to facilitate their spreading. Interestingly, the genes in the ceRNA networks of EBV, HCMV, HSV2, and MCV are found to be enriched for focal adhesion. Notably, EBV, HCMV, and HSV2 belong to the Herpesvirus family which has been reported to activate focal adhesion kinase (FAK), critical for the entry of Herpesviruses into the target cell (Cheshenko et al., 2005). The genes in the ceRNA networks of HCMV, HIV1, HSV1, and HSV2 are enriched for Platelet activation, signaling and aggregation. Enriched pathways in the ceRNA networks for each of the 10 viruses used in this study can be viewed from the database url http://gyanxet-beta.com/humanvice.

### Identification of viral miRNA targets on host lncRNAs from PAR-CLIP in virus infected cells

We identified viral miRNA targets on host lncRNA loci from AGO PAR-CLIP dataset in EBV, HCMV, and KSHV infected human cells. Datasets of PAR-CLIP performed in EBV B95-8-infected LCLs was collected from NCBI GEO database under GEO accession id GSE41437. We identified 34 targets of 16 different EBV miRNAs on 29 different human lncRNA loci. Similarly, we found 1 HCMV miRNA target on 1 human lncRNA loci from AGO PAR-CLIP dataset from HCMV infected primary human fibroblast cells (GSE33584). From the PAR-CLIP dataset on two latently KSHV infected PEL cell lines, BCBL-1 and BC-3 (GSE41357), we identified 69 targets of 16 different KSHV miRNAs on the genomic loci of 55 different human lncRNA loci (data downloadable from http://gyanxet-beta.com/humanvice). These results suggest that host lncRNAs are likely to be targeted by viral miRNAs.

### Predicted ceRNA activity mediated by host and viral miRNAs in kaposi's sarcoma associated herpesvirus (KSHV) infected cells from PAR-CLIP dataset

**Figure 3:**
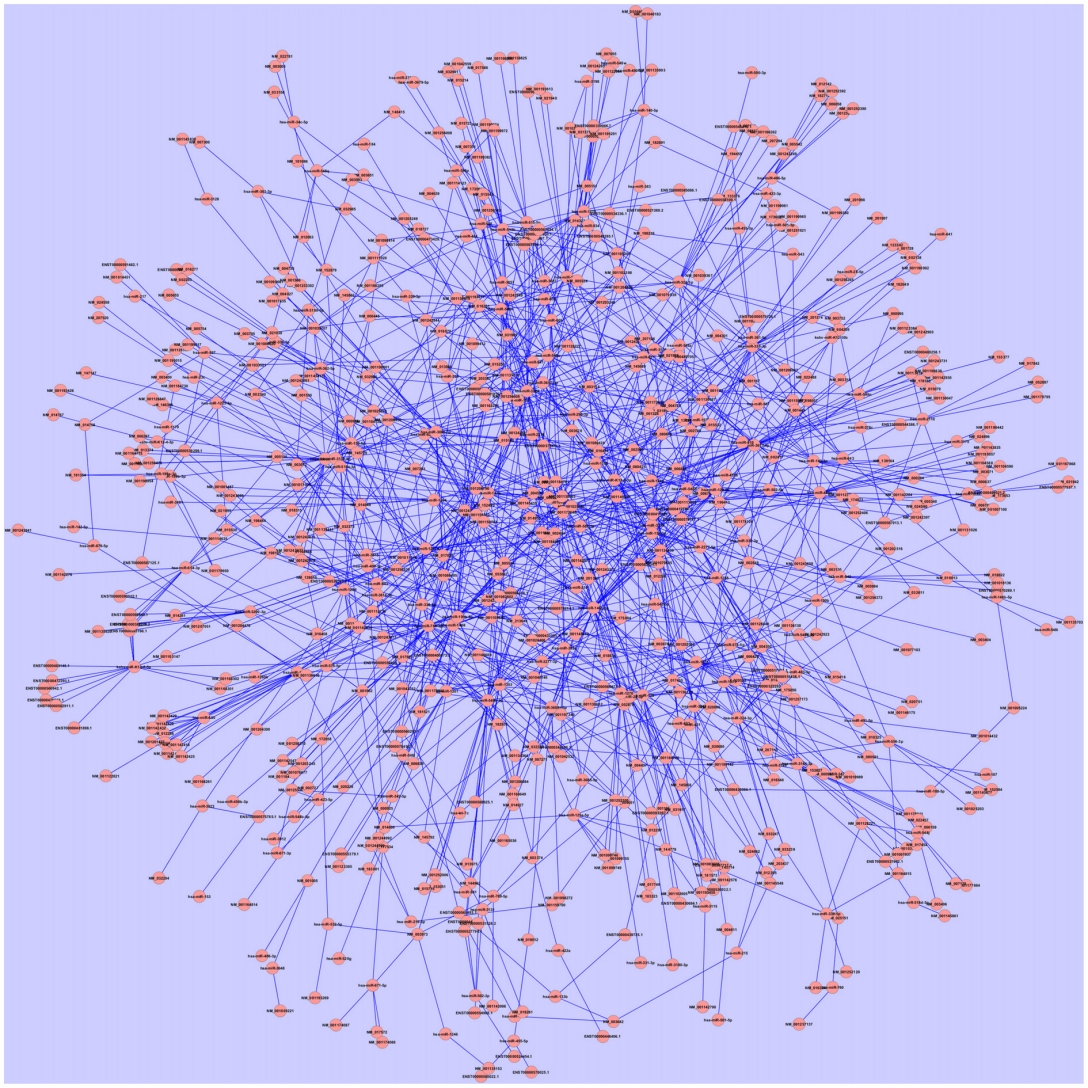
**The ceRNA network in KSHV infected PEL cells incorporating predicted cellular protein-coding, lncRNA and circRNA targets of both host cellular and viral miRNAs identified from AGO PAR-CLIP data**. The network comprises of 762 protein-coding and 144 non-coding targets of 1717 distinct human miRNAs and 19 distinct KSHV miRNAs. As analyzed by cytoscape, the average number of neighbors is 3.799 and network centralization is 0.04.

**Figure 4:**
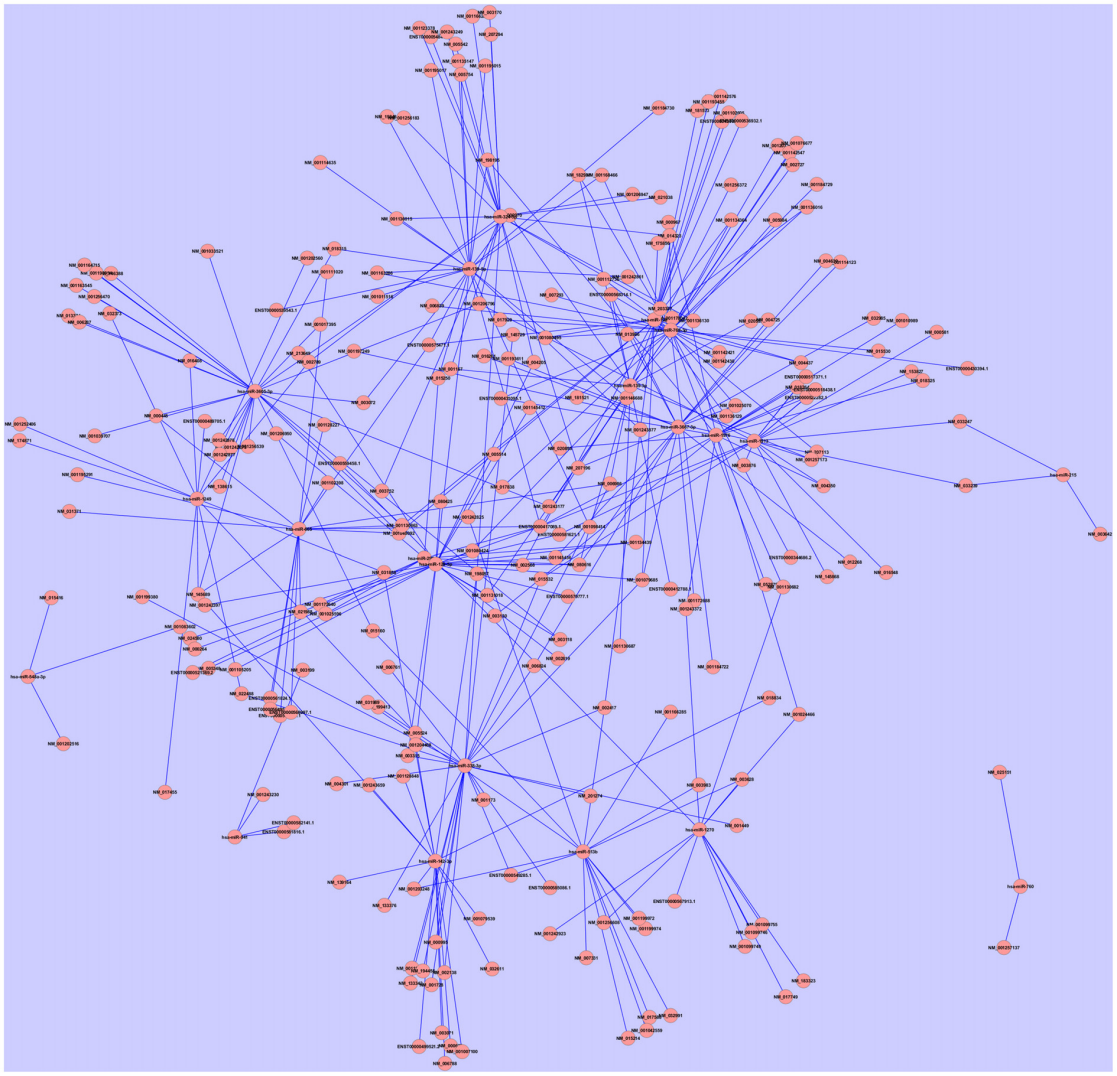
**The ceRNA network around the 24 host immune response associated genes (listed in Table 2)**. This network comprises of 246 transcripts (217 protein-coding and 29 non-coding), targeted by 21 distinct host miRNAs, which were found to have potential ceRNA effects on 24 host immune response associated genes. Compared to the whole ceRNA network in KSHV infected PEL cells (Figure 3), this network has increased centralization index 0.112.

Kaposi's sarcoma associated herpesvirus (KSHV) or Herrpesvirus 8 (HHV-8) is an oncovirus that causes Kaposi's sarcoma and PEL in human. We identified viral and host miRNA targets from PAR-CLIP datasets in KSHV infected PEL cells that may act as competing endogenous RNA (ceRNA). Computational analysis identified 762 protein-coding and 144 non-coding targets of 1717 distinct human miRNAs and 19 distinct KSHV miRNAs. We developed the whole predicted ceRNA network consisting of host mRNAs, lncRNAs and circRNAs targeted by human and viral miRNAs in KSHV infected cells (Figure 3). We did GO analysis on the set of protein-coding targets of the host and viral miRNAs using Gorilla GO enrichment analysis tool (Eden et al., 2009). Importantly, we got enrichment for the GO cellular component phagocytic vesicle membrane. We further studied the potential miRNA mediated regulation of host immune response associated genes as identified from the AGO PAR-CLIP dataset on KSHV infected PEL cell lines. From a list of 1535 immune response associated genes downloaded from InnateDB (Breuer et al., 2013), 24 genes were found to be targeted by miRNAs in KSHV infected PEL cell line. Table 2 lists the host immune response related genes targeted by miRNAs in KSHV infected PEL cells. We looked into the ceRNA network around these 24 host immune response associated genes. A total of 246 transcripts (217 protein-coding and 29 non-coding), targeted by 21 distinct host miRNAs, were found to have potential ceRNA effects on 24 host immune response associated genes. Statistical analysis showed that the transcripts potentially targeted by miRNAs in KSHV infected human PEL cells were enriched for having a ceRNA effect on the host immune response associated genes (*p*-value < 0.01, calculated using hypergeometric test). The ceRNA network consisting of only host immune response related targets of host and viral miRNAs is shown in Figure 4.

**Table 2:** **Host immune response associated genes targeted by miRNAs in KSHV infected PEL cells**.

**Gene name**	**Gene symbol**	**Transcript accession**	**Targeting miRNA name**
Annexin A11	ANXA11	NM_145868	hsa-miR-1913
Amyloid beta (A4) precursor protein	APP	NM_001136016	hsa-miR-128
Basigin (Ok blood group)	BSG	NM_001728	hsa-miR-338-3p
Complement component 4A (Rodgers blood group)	C4A	NM_007293	hsa-miR-769-3p
Caspase 3, apoptosis-related cysteine peptidase	CASP3	NM_032991	hsa-miR-513b
CD4 molecule	CD4	NM_001195017	hsa-miR-139-5p
CASP8 and FADD-like apoptosis regulator	CFLAR	NM_001202516	hsa-miR-548a-3p
ELK1, member of ETS oncogene family	ELK1	NM_001114123	hsa-miR-3667-3p
Glucosamine (UDP-N-acetyl)-2-epimerase/N-acetylmannosamine kinase	GNE	NM_001128227	hsa-miR-605
Major histocompatibility complex, class I, B	HLA-B	NM_005514	hsa-miR-129-5p
Itchy E3 ubiquitin protein ligase	ITCH	NM_001257137	hsa-miR-760
Integrin, beta 1 (fibronectin receptor, beta polypeptide, antigen CD29 includes MDF2, MSK12)	ITGB1	NM_133376	hsa-miR-338-3p
Promyelocytic leukemia	PML	NM_033247	hsa-miR-215
Proteasome (prosome, macropain) subunit, alpha type, 4	PSMA4	NM_002789	hsa-miR-324-5p
Proteasome (prosome, macropain) 26S Subunit, non-ATPase, 12	PSMD12	NM_174871	hsa-miR-1249
Ribosomal protein L3	RPL3	NM_000967	hsa-miR-1976
Superoxide dismutase 2, mitochondrial	SOD2	NM_001024466	hsa-miR-1270
Secreted protein, acidic, cysteine-rich (osteonectin)	SPARC	NM_003118	hsa-miR-296-3p
Serglycin	SRGN	NM_002727	hsa-miR-769-3p
Transcription factor 4	TCF4	NM_003199	hsa-miR-941
Tissue factor pathway inhibitor (lipoprotein-associated coagulation inhibitor)	TFPI	NM_006287	hsa-miR-3605-3p
TSC22 domain family, member 3	TSC22D3	NM_198057	hsa-miR-142-3p
X-box binding protein 1	XBP1	NM_001079539	hsa-miR-142-3p
X-linked inhibitor of apoptosis	XIAP	NM_001167	hsa-miR-139-3p

### Predicted ceRNA activity in human immunodeficiency virus 1 (HIV1) infected peripheral blood mononuclear cells (PBMC)

We checked for the predicted targets of host and viral miRNAs expressed in HIV1 infected human Peripheral blood mononuclear cells (PBMC). From a dataset of miRNA expression profiling in HIV1 infected PBMC (GSE44332), out of 339 host miRNAs expressed in HIV1 infected PBMCs, we found 87 miRNAs potentially targeting 1370 human immune response associated gene transcripts out of total 1535 immune response associated gene transcripts listed in InnateDB (Breuer et al., 2013). Our analysis further revealed extensive ceRNA networks around these host immune response associated transcripts in HIV1 infected PBMC cells (data downloadable from http://gyanxet-beta.com/humanvice). This suggested an immensely complex regulatory circuit around host immune response associated genes in HIV1 infected PBMCs. As the presence of ceRNAs was known to have a diluting effect on the miRNA mediated regulation of a gene, the extensive predicted ceRNA network around the immune response related genes targeted by miRNAs in HIV1 infected PBMCs should have significant effects on fine tuning of their expressions in these cells. APOBEC family genes have been shown to inhibit HIV1 replication as part of the innate immune system. We looked into the miRNA target network around APOBEC family genes. Many of the miRNAs expressed in HIV1 infected PBMCs were predicted to target APOBEC family genes including APOBEC1, APOBEC2, APOBEC3G, and APOBEC4 (see Table 3) and they have an extensive potential ceRNA network around them. The presence of the ceRNA network around APOBEC family genes suggests of an alternative strategy of the host immune system toward antiviral defense.

**Table 3:** **Targets of miRNAs expressed in HIV infected PBMCs on human APOBEC family genes**.

**Gene symbol**	**Transcript accession (RefSeq)**	**Targeting miRNAs**
APOBEC2	NM_006789	hsa-miR-324-3p,hsa-miR-329,hsa-miR-107,hsa-miR-378,hsa-miR-770-5p,hsa-miR-508-3p,hsa-miR-508-3p
APOBEC3G	NM_021822	hsa-miR-520g
APOBEC3D	NM_152426	hsa-miR-508-5p,hsa-miR-125a-5p,hsa-miR-32,hsa-miR-423-5p,hsa-miR-1,hsa-miR-206,hsa-miR-129-5p,hsa-miR-129-5p,hsa-miR-107,hsa-miR-210,hsa-miR-512-5p,hsa-miR-615-3p
APOBEC4	NM_203454	hsa-miR-125a-5p,hsa-miR-298,hsa-miR-22,hsa-miR-142-5p,hsa-miR-199a-3p,hsa-miR-301b,hsa-miR-372,hsa-miR-372,hsa-miR-494,hsa-miR-496,hsa-miR-520g,hsa-miR-93,hsa-miR-484
APOBEC1	NM_001644	hsa-miR-329,hsa-miR-526a
APOBEC3A	NM_145699	hsa-miR-372,hsa-miR-520g,hsa-miR-93,hsa-miR-129-5p,hsa-miR-129-5p,hsa-miR-433
APOBEC3F	NM_145298	hsa-miR-508-5p,hsa-miR-1197,hsa-miR-125a-5p,hsa-miR-298,hsa-miR-22,hsa-miR-32,hsa-miR-423-5p,hsa-miR-671-5p,hsa-miR-1,hsa-miR-206,hsa-miR-450b-5p,hsa-miR-494,hsa-miR-527,hsa-miR-760,hsa-miR-875-3p,hsa-miR-93,hsa-miR-129-5p,hsa-miR-208a,hsa-miR-208b,hsa-miR-299-3p,hsa-miR-484,hsa-miR-296-3p,hsa-miR-342-3p,hsa-miR-210,hsa-miR-512-5p,hsa-miR-615-5p,hsa-miR-485-3p
APOBEC3C	NM_014508	hsa-miR-125a-5p,hsa-miR-423-5p,hsa-miR-1,hsa-miR-206,hsa-miR-875-3p,hsa-miR-875-3p,hsa-miR-107
APOBEC3H	NM_001166003, NM_001166002, NM_181773	hsa-miR-372,hsa-miR-520g,hsa-miR-93
APOBEC3H	NM_001166004	hsa-miR-770-5p

### Utility of humanViCe

**Figure 5:**
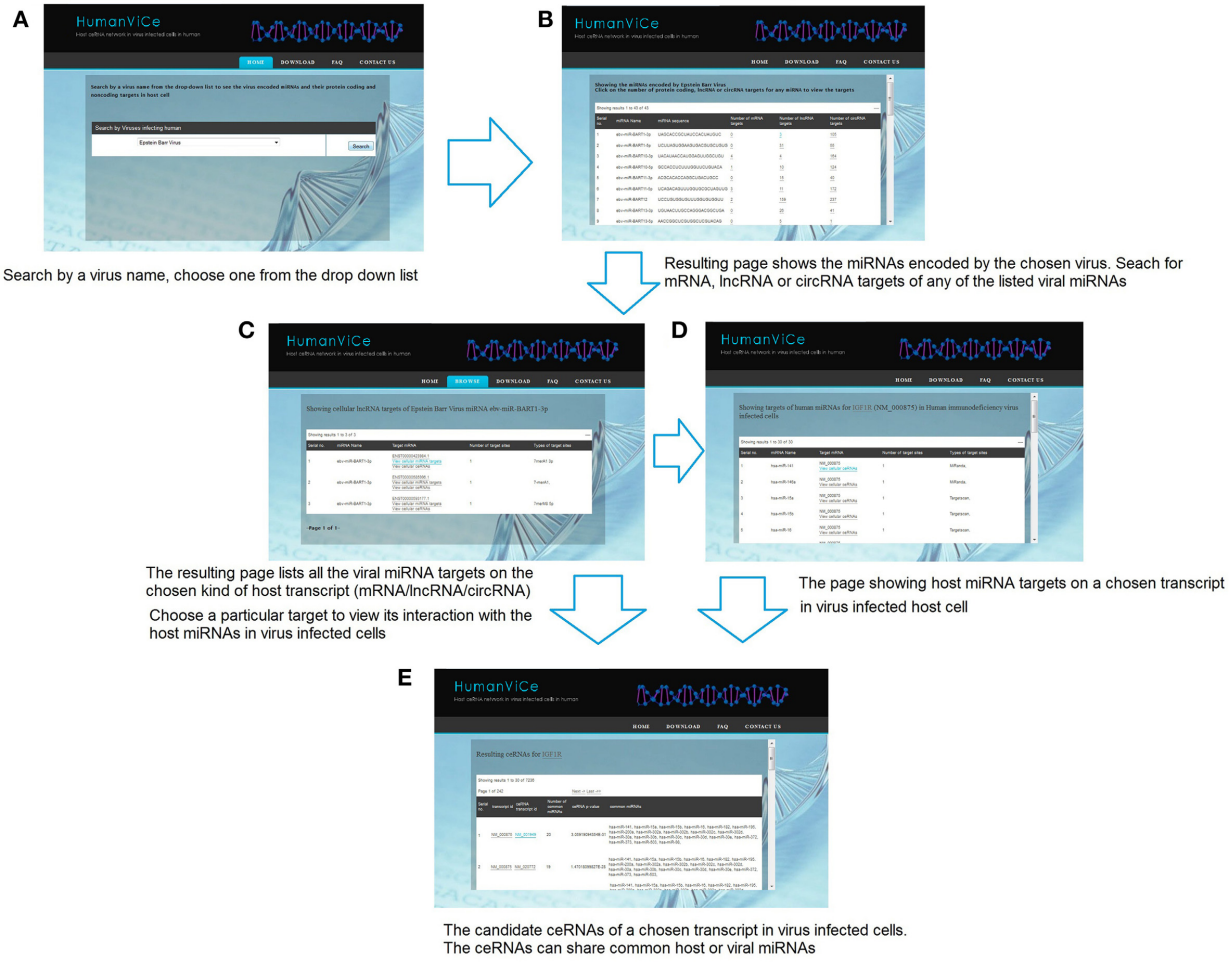
**The navigation of HumanViCe is depicted**. **(A)** Users can search by a virus name. **(B)** Resulting page shows the list of miRNAs encoded by the chosen virus along with the number of mRNA, lncRNA and circRNA targets of each of the viral miRNAs. Searching for targets of a particular transcript type (mRNA, lncRNA or circRNA) for a particular viral miRNA from the list results in **(C)** a page listing all the targets of the chosen type of the chosen viral miRNA. **(D)** The users can search for host miRNA targets on a particular transcript from the list. The resulting page includes only interactions with the host miRNAs those are expressed cells infected with the particular virus. **(E)** The users can search for potential ceRNAs of a chosen host transcript. The ceRNAs may share common host or viral miRNAs expressed in virus infected cells.

Users can browse for viral miRNA targets on host protein-coding and non-coding genes by choosing a particular virus. The resulting page lists miRNAs encoded by the chosen virus along with its mRNA, lncRNA, and circRNA targets in host cells. Users can view details of the targets by choosing a particular viral miRNA. There is also provision for checking the host cellular miRNA targets on a particular viral miRNA target transcript. The users can check the potential ceRNAs for a chosen transcript. The resulting page shows the potential ceRNA candidates that share one or more common cellular or viral miRNA(s), sorted by their probability (*p*-value) to act as ceRNA to the chosen transcript. The list of ceRNAs is sorted by the number of shared miRNAs. The miRNA targets for a particular lncRNA, mRNA, or circRNA can also be browsed in our database by choosing a particular lncRNA/mRNA/circRNA from the ceRNA list. The usage of HumanViCe is described in Figure 5. The users can download the results of pathway enrichment analysis for the genes comprising the ceRNA networks for each of the 10 viruses included in HumanViCe after searching by a particular virus name.

## Discussions

It has been observed that both cellular and viral miRNAs (miRNAs encoded by virus) play important roles on host-viral interaction. The complex cross-talk between host and viral miRNAs and their cellular and viral targets form the environment for viral pathogenesis. The current study is aimed at unraveling the cross-talk-network of the viral and host miRNA targets in virus infected cells in human.

In recent years, it has been observed that other than protein-coding transcripts, cellular miRNAs can also target other non-coding RNA like lncRNA and circRNA. Cellular transcripts (mRNAs, pseudogenes, lncRNAs, or circRNAs) sharing target sites for one or more common miRNAs compete with each other for the limited pool of cellular miRNAs and thus affect the competing RNA's level, a phenomenon known as ceRNA effect. This ceRNA effect is known to play significant roles in important biological processes including many disease pathogenesis as crucial new determinants of gene expression regulation. As viral miRNAs have already been reported to interact with host cellular factors, and viral miRNAs may have potential interaction with cellular non coding RNAs as well. Furthermore, it is highly likely that viral miRNAs may exploit the host gene regulatory network via the ceRNA effect. A virus exploiting the cellular miRNA mediated gene regulatory network via ceRNA effect has been already reported. In a previous study Cazalla and his group reported that viral U-rich non-coding RNAs called HSUR expressed in primate virus HVS infected T cells are able to bind to three host miRNAs resulting in a striking alteration of the cellular levels of one of these miRNAs, miRNA-27, which in turn may impair the regulation of the cellular targets of that miRNA in HVS infected cells. In our study, we identified viral miRNA targets on host lncRNA loci from AGO PAR-CLIP dataset in EBV, HCMV, and KSHV infected human cells. circRNAs are reported to contain extensive miRNA binding sites for effective miRNA sponge activity. From our study we identified some circRNAs containing large number of binding sites for some viral miRNAs; e.g., human circRNA transcript of ANKRD11 (circRNA id: hsa_circ_002048) was found to bear 36 sites for EBV encoded miRNA ebv-miR-BART20-5p. Our computational analysis identified many protein-coding and non-coding transcripts targeted by common viral or host miRNAs that points to a possible ceRNA effect in the virus infected cells. When we looked into the predicted ceRNA networks for all the 10 viruses used in current study, we found genes enriched for cellular signaling pathways commonly exploited by viruses. The most common enriched pathways for all the viruses include axon guidance, NOTCH signaling pathway, MAPK signaling pathway, Wnt signaling pathway. These pathways are related to cell differentiation state, gene transcription and intracellular signaling and prone to be manipulated by viruses after infection. Also the enrichment of pathways related to viral entry, replication and virulence was observed. The genes in the ceRNA networks of EBV, HCMV, HIV, HSV1, HSV2, and KSHV were found to be enriched for endocytosis. The genes in the ceRNA networks of EBV, HIV, HSV1, HSV2, and KSHV were found to be enriched for membrane trafficking. ceRNA networks of EBV, HCMV, HSV2, and MCV were found to be enriched for focal adhesion. The genes in the ceRNA networks of HCMV, HIV, HSV1, and HSV2 were enriched for platelet activation, signaling and aggregation.

We especially looked into the possible ceRNA networks around human immune response associated genes in KSHV and HIV infected cells. Our analysis suggested that transcripts potentially targeted by miRNAs in both KSHV infected human PEL cells and HIV infected PBMCs were enriched for having a ceRNA effect on the host immune response associated genes. Furthermore, we identified a vast number of non-coding transcripts playing as potential ceRNAs to the immune response associated genes in these virus infected cells. Importantly, we identified seed matched targets sites of 87 miRNAs expressed in HIV infected PBMCs on 1370 host immune response associated genes out of the total 1535 genes listed in InnateDB. Furthermore, extensive predicted ceRNA network was identified around these host immune response associated genes, which suggested of a complex regulatory circuit around host immune response associated genes in HIV infected PBMCs. Working toward understanding the regulatory effect of the ceRNA network in HIV infected cells will be our future direction.

We developed HumanViCe, a repository of the putative viral and host (human) miRNA interaction with cellular protein-coding RNA, lncRNA, and circRNAs in virus infected cells in human. Potential ceRNAs in different virus infected cells can be browsed in HumanViCe, where we have also calculated a ceRNA score (measured using the number of shared miRNAs between a pair) for each potential ceRNA pair to show the likelihood for them to act as ceRNAs. Put together, HumanViCe can be a very useful tool for researchers working on host-virus interactions to understand the dynamics of host and viral miRNA mediated regulations in virus infected cells.

## Database availability

HumanViCe is available freely from http://gyanxet-beta.com/humanvice

### Conflict of interest statement

The authors declare that the research was conducted in the absence of any commercial or financial relationships that could be construed as a potential conflict of interest.
